# Oropharyngeal dysphagia management and informed consent: a survey of speech-language pathologists’ practice patterns when recommending modified texture diets

**DOI:** 10.3389/fresc.2025.1520240

**Published:** 2025-05-09

**Authors:** Matthew G. Ward, Angela S. Bowman

**Affiliations:** ^1^ SA Swallowing Services, Nashville, TN, United States; ^2^ Department of Health and Human Performance, Middle Tennessee State University, Murfreesboro, TN, United States

**Keywords:** dysphagia, thickened liquids, modified diet, speech-language pathologist, informed consent

## Abstract

**Purpose:**

The modification of diet textures and liquid viscosity represents the primary form of management of oropharyngeal dysphagia (OD) by speech-language pathologists (SLPs). Despite the ubiquitous use of modified texture diets (MTDs) to prevent aspiration in an attempt to prevent pneumonia, there is no convincing evidence that consumption of MTDs protects individuals with dysphagia from developing pneumonia. Furthermore, informed consent is required for the prescription of MTDs. To date, no study has investigated if practicing SLPs know the risks associated with MTDs, consider the risks when making clinical decisions, and disclose those risks to their patients.

**Method:**

Thirteen negative health outcomes associated with MTDs were identified in the research literature. A web-based survey was created and distributed. Participants were asked to identify known risks associated with MTDs, how often they considered the risks associated with MTDs before recommending them, and how often they informed patients with OD of the known risks associated with MTDs.

**Results:**

Only 6.3% of the SLPs surveyed identified all thirteen listed health risks associated with MTDs, and greater than one in five respondents (*n* = 55; 21.7%) were unable to select even one known risk. Seventy percent (*n* = 140) of participants indicated that they “almost always” weigh the risks associated with drinking thickened liquids, and fifty-four percent of respondents (*n* = 108) specified that they “almost always” weigh the risks associated with consuming modified texture solids. Less than half of the participants (*n* = 99; 49.7%) stated that they “almost always” inform the patient of the risks associated with thickened liquids and 39.9% (*n* = 79) indicated that they “almost always” inform patients of the risks associated with altered texture solids.

**Conclusions:**

Participants demonstrated poor overall knowledge of the hazards associated with MTDs, limited consideration of known risks of MTDs, and suboptimal levels of disclosure of the risks associated with MTDs to patients with OD.

## 1 Introduction

A central pillar of oropharyngeal dysphagia (OD) management, by speech-language pathologists (SLPs), is the use of modified texture diets (MTDs) to mitigate pneumonia risk. MTDs encompass any combination of altering food consistency, increasing liquid viscosity, or restricting the manner of oral eating and drinking (1–3). For over 50 years, SLPs attempted to prevent or decrease aspiration in the hopes of reducing the rate of pneumonia from aspiration among patients with OD (4, 5). However, over the past 25 years, the body of evidence in the scientific literature is clear that prandial aspiration alone is insufficient for the development of pneumonia, and there is no strong evidence to support the use of MTDs to prevent pneumonia (6–14). Further, MTDs are associated with significant negative impacts on health and quality of life (QOL) (1, 15–22). Despite the inability to provide meaningful pneumonia prevention and despite the consensus that eating and drinking MTDs can result in multiple negative health outcomes, MTDs currently represent the standard of clinical care provided by SLPs who diagnose and treat individuals with dysphagia (2, 3, 20, 23, 24).

While a relatively new concept, only arising in the latter half of the 20th century, informed consent initially served to protect individual autonomy when participating in research (25). However, informed consent is also key to protecting patient rights in clinical settings—allowing for increased patient autonomy and improvements in shared decision making among patients and medical professionals (26). Thus, informed consent is required for any clinical intervention. As with other medical interventions, changes to an individual's diet are not without risk, and informed consent is required for the use of MTDs for the management of dysphagia (27, 28). To be informed, an individual:

“…must be given sufficient information in a way that they can understand about what the treatment involves, including the potential benefits and harms, whether there are reasonable alternative treatments, and what will happen if treatment does not go ahead … This requires consideration of the quality of evidence that an intervention will be successful in achieving meaningful endpoints that are important to a patient” (27, p. 2).

It has been suggested that SLPs—as a body of practitioners—may not be fully aware that informed consent is required when prescribing MTDs (29). If SLPs are merely unaware of the need for informed consent, then the solution to the problem of informed consent could be relatively simple. By providing SLPs with updated guidance that disclosure of all risks is required for the use of MTDs in medical settings, the issue could be improved.

However, O’Keeffe et al. (29), hypothesize that the near universal use of MTDs without informed consent also arises from misconceptions of the efficacy of MTDs to prevent pneumonia and its consequences. This barrier to effective treatment is more insidious than the previously discussed misapprehension of the need to provide a full accounting of the possible health outcomes that result from the treatment of OD with MTDs, because patient autonomy and shared decision making are predicated on an SLP's knowledge of the harms and benefits associated with MTDs. In other words, an SLP must have sufficient education and training so that a clinician would be able to explain to an individual with dysphagia all of the possible positive and negative outcomes associated with any form of treatment or non-treatment. To date, no survey of clinical practice patterns has investigated SLPs’ knowledge of health outcomes associated with MTDs.

While SLPs have extensive training in diagnosing and rehabilitating OD at the graduate level, there is no requirement that SLPs receive training on the impact that MTDs have on body systems and whole-body homeostasis (30, 31). Over the past twenty-five years, multiple well-designed and replicated studies have illuminated numerous negative health outcomes associated with MTDs (1, 7, 22, 32–36). Due to the known complications with MTDs and the lack of any requirement for SLP training regarding the hazards related to MTDs, three questions remain. First, do SLPs know the risks associated with consuming MTDs? Second, do SLPs consider the risks associated with consuming MTDs? Third, do SLPs disclose those risks to their patients?

## 2 Methods

Using Qualtrics, a pilot e-survey was completed and disseminated to a small group of experienced SLPs who routinely evaluate and treat individuals with OD. The feedback provided allowed for hypothesis development and refinement of the questions that were ultimately used for the final distributed survey (see Supplementary Appendix A for a complete list of all survey items).

This manuscript uses data from a small subset of questions that were a part of a larger survey intended to investigate what factors SLPs find most important when making treatment recommendations for patients with dysphagia. For the purposes of this manuscript, the questions selected sought to provide participants with concrete examples of known risks that are associated with MTDs. The complications associated with MTDs have been studied for over two decades by a wide variety of researchers and frontline clinicians, and the published peer-reviewed evidence base is abundant. However, rather than attempting to provide an exhaustive inventory of all possible risks associated with MTDs, the authors endeavored to provide SLP participants with a group of known complications that directly impact the health and well-being of the individuals with OD.

### 2.1 Distribution and recruitment

Participants were recruited through a variety of electronic means. A link to the survey was provided in the American Speech Language Hearing Association Special Interest Groups 13 (Swallowing & Swallowing Disorders). Participants were also recruited via survey link in the following Facebook communities: The Curious SLP, Curious SLP Admin Group, FEES and MBS Discussion Group, Medical SLP Forum, SLPs in SNFs, Adult Rehab Speech Therapy, Head and Neck Cancer SLP Group, Dysphagia ProConnect, SLP Medical Research Group, Association of Independent FEES Providers, FEES for SLPs, D3, Swallowing and Swallowing Disorders Journal Club. Lastly, the authors sent a link to the survey via email to professional contacts who were also able to forward the link to other individuals.

Survey enrollment was voluntary, and no compensation or incentives were offered to participants. The following message was displayed with the anonymous survey link: “We are conducting evidence-based research on clinical decision making in dysphagia practice. Your participation will help us to better understand clinical practice patterns for those who diagnose and treat individuals with dysphagia. You will be answering questions about your typical practice when evaluating dysphagia. You will be asked to answer a series of demographic and clinical questions. Answers are completely anonymous, and the survey should take between 10 and 20 min to complete. Thank you!”

The survey was active from March 16, 2022 to April 30, 2022. Respondents were not required to complete the entire survey in one session, and they were also able to return to previously answered questions to change answers as needed. The survey could be accessed on a computer or mobile device (e.g., smart phone or tablet). Completion of the entire survey was not required for participant answers to be included in the data analysis. Respondents were allowed only to submit a single survey. To discourage participants from filling out multiple surveys, the first demographic question was, “Have you taken this survey before?” A “yes” response prevented further participation in the study.

### 2.2 Participants

There were two inclusion criteria for this survey. Participants must be 18 years of age or older, and participants must be practicing SLPs who diagnose and treat OD. A total of 327 respondents opened and initiated the survey. A single participant answered that they were not 18 years old or older, and their responses were excluded from analysis. The final sample included responses from 326 participants. With the exception of the single participant who indicated that they were not 18 years or older, no data was excluded from analysis.

Table 1 provides race and ethnicity data for SLPs from the American Speech-Language-Hearing Association's (ASHA) Member and Affiliate Profile (AHSA, 2021). A total of 250 participants reported their race; the majority of the participants (*n* = 225; 90%) reported their best described race as white and no other group represented more than 4%. The corresponding demographics for survey participants are listed alongside data from ASHA's Member and Affiliate Profile, and the demographic representation of the participants was consistent with the overall population of healthcare-based SLPs. After dispensing with demographic questions, respondents were asked nineteen survey questions about OD evaluation and treatment.

**Table 1:** Demographics: race & ethnicity.

Race	ASHA members (%) (*n* = 168,359)	Participants (%) (*n* = 323)
American Indian or Alaska Native	0.3	0.3
Asian	3.0	3.4
Black or African American	3.7	1.8
Native Hawaiian or other Pacific Islander	0.2	0
White	91.4	89.5
Multiracial	1.5	1.32
Prefer not to answer	n/a	1.6
Ethnicity	ASHA Members (%) (*n* = 168,359)	Participants (%) (*n* = 310)
Hispanic or Latino	6.3	4.2
Not Hispanic or Latino	93.7	93.9
Prefer not to answer	n/a	1.9

American Speech-Language-Hearing Association. (2022). 2021 Member and affiliate profile. https://www.asha.org.

Tables 2–5 delineate participant: age, primary employment setting, experience as an SLP, and experience treating individuals with dysphagia. The age categories for the survey were: 24 or younger, 25–34, 35–44, 45–54, and 65 years & up, with the majority of participants (69.4%) selecting 44 years or younger as shown in Table 2. The majority of clinicians reported working in acute hospitals (41.2%) and Table 3 details the breakdown of primary work settings. Professional experience as an SLP, calculated in years, and years spent treating individuals with OD are delineated in Tables 4, 5.

**Table 2:** Demographics: Age .

Age	ASHA members (%) (*n* = 177,061)	Participants (%) (*n* = 308)
34 and younger	28.1	36.0
35–44	28.7	33.7
45–54	22.4	19.5
55–64	12.8	8.1
64 and older	8.0	2.6

American Speech-Language-Hearing Association. (2022). 2021 Member and affiliate profile. https://www.asha.org.

**Table 3:** Demographics: primary employment setting.

Primary employment setting (health care)	ASHA members (%) (*n* = 66,743)	Participants (%) (*n* = 313)
Hospital	31.0	41.2
Skilled Nursing Facility	18.3	22.7
Other Residential HCF	3.6	6.4
All other settings	47.2	29.8

American Speech-Language-Hearing Association. (2022). 2021 Member and affiliate profile. https://www.asha.org.

**Table 4:** Experience as SLP.

Years	Frequency (*n*)	Percent (%)
<1	12	4.9
1–5	49	20.2
6–10	55	22.6
11–20	66	27.2
21+	61	25.1
Total	243	100.0

**Table 5:** Experience as SLP treating dysphagia.

Years	Frequency (*n*)	Percent (%)
<1	17	7.0
1–5	48	19.8
6–10	56	23.0
11–20	69	28.4
21+	53	21.8
Total	243	100.0

### 2.3 Training

In the United States, the majority of SLPs who work in a medical setting have a master's degree, and 230 participants (95.0%) indicated that the highest degree they earned was at the master's level. Only 4.9% of respondents indicated that they had attained a clinical or research doctorate. While specific training regarding the relationship between malnutrition and dehydration secondary to OD is not a required part of educational curriculum for SLPs, 177 individuals (75.0%) indicated that they had received formal training regarding the relationship between OD and malnutrition and dehydration—with only 59 participants (25.0%) indicating that they received no formal training.

### 2.4 Survey items

This investigation was part of a larger survey of SLP knowledge and training regarding the relationship of malnutrition, dehydration, and pneumonia that occur secondary to OD. Hence, not all questions from the original survey were used for this current study. For this examination, questions 7–12 and 14 & 15 were used, and 253 participants partially or fully completed these 8 questions.

In questions 7 & 8, participants were asked if they recommended thickened liquids or altered solid diet textures when needed for which they could answer “yes” or “no”. For questions 8–10, participants could select “yes”, “no”, or “I don’t know” to questions about their background knowledge of dysphagia, malnutrition, and dehydration. In questions 11 & 12 participants were asked to identify all known risks associated with thickened liquids or altered diet textures from a list of 15 possible answers: “malnutrition”, “dehydration”, “respiratory infection”, “poor recovery from illness”, “constipation”, “urinary tract infection”, “slow digestion”, “interfere with medication absorption”, “decreased quality of life”, “constant feeling of thirst”, “none of the items apply”, or “I don’t know”. Lastly, in questions 14 & 15 participants were asked to select how frequently they weighed the risks of MTDs to those of non-treatment and how frequently participants informed their patients of the risks associated with MTDs. Participants could choose among the following answers for each question: “almost always (90% or more)”, “very frequently (60%–89%)”, “occasionally (40%–59%)”, or “rarely (10%–39%)”, or “almost never (less than 10%)”.

### 2.5 Data analysis

Descriptive statistics (i.e., frequencies, percentages, means, median, and mode) were used to report demographic data and to explore patterns of practice. Participants were asked two objective questions about known risks of MTDs. Respondents were free to choose any, and all, risks they knew to be associated with altered texture solids and thickened liquids, and they were also free to choose “none of the items apply” or “I don’t know”.

Of the 12 responses listed as possible side effects of modifying solid textures, four are well-supported in the research literature: malnutrition (17–19, 21), dehydration (37), poor recovery from illness (38, 39), decreased QOL (40, 41). For side effects of thickened liquids, 9 of the 12 possible responses qualify as known possible side effects that are supported in the research literature: dehydration (1, 42), respiratory infection (43, 44), poor recovery from illness (38, 39), constipation (1), urinary tract infection (45), slow digestion (1), interference with medication absorption (46, 47), constant feeling of thirst (1, 45, 48), and decreased QOL (40, 41).

Thus, for modified texture solids, four correct selections were available: malnutrition, dehydration, poor recovery from illness, and decreased QOL. For thickened liquids, there were nine options that could be chosen: dehydration, respiratory infection, poor recovery from illness, constipation, urinary tract infection, slow digestion, interference with medication absorption, constant feeling of thirst, decreased QOL. Combining the two lists, for thickened liquids and for modified texture solids, resulted in a total of 13 negative health outcomes that could have been selected by participants.

### 2.6 Ethics

Information obtained in this survey could not be linked to the participants, and the survey was approved by the Institutional Review Board at Middle Tennessee State University (See Supplementary Appendix B for IRB approval letter).

## 3 Results

### 3.1 Malnutrition & dehydration

While malnutrition and dehydration are two major and known complications of OD, they can also result from using MTDs, which are prescribed to decrease the risk of aspiration and pulmonary complications. Indeed, 96% of respondents indicated that they recommended altered texture solids when needed, and 93.5% of clinicians recommend the use of thickened liquids when needed. When asked if there was a known relationship between the consumption of altered texture solids and malnutrition, 93.5% of clinicians indicated that there was a known relationship, and 98.5% of clinicians answered that there is a relationship between dehydration and consuming MTDs. Furthermore, 190 participants (75.1%) selected dehydration as a known risk of consuming thickened liquids, and 183 respondents (72.3%) indicated that malnutrition was a known risk of consuming modified texture solids.

Participants were asked if they personally weighed the known risks of MTDs vs. the known risks associated with aspiration (see Tables 6, 7)—with 70% (*n* = 140) selecting that they “almost always (90% or more)” weigh the known risks of aspiration against those of consuming thickened liquids and 54% (*n* = 108) of clinicians indicated that they “almost always (90% or more)” weigh the known risks associated with altered solid textures vs. known harms associated with aspiration prior to recommending them. Further, only 49.7% (*n* = 99) of participants specified that they “almost always (90% or more)” inform the patient of the known risks associated with consuming thickened liquids, and only 39.9% (*n* = 79) of SLPs indicated that they “almost always (90% or more)” inform the patient of the known risks associated with altered solid textures (see Tables 8, 9).

**Table 6:** “before recommending thickened liquids, I weigh the known risks of aspiration against the known risks of consuming thickened liquids”.

Response	Frequency (*n*)	Percent (%)
Almost Always (90% or more)	140	70.0
Very Frequently (60%–89%)	46	23.0
Occasionally (40%–59%)	10	5.0
Rarely (10%–39%)	4	2.0
Total	200	100.0

**Table 7:** “before recommending altered diet textures, I weigh the known risks of aspiration against the known risks of consuming altered diet textures”.

Response	Frequency (*n*)	Percent (%)
Almost Always (90% or more)	108	54.0
Very Frequently (60%–89%)	64	32.0
Occasionally (40%–59%)	19	9.5
Rarely (10%–39%)	7	3.5
Almost Never (less than 10%)	2	1.0
Total	200	100.0

**Table 8:** “before recommending thickened liquids, I inform the patient of the possible risks associated with thickened liquids”.

Response	Frequency (*n*)	Percent (%)
Almost Always (90% or more)	99	49.7
Very Frequently (60%–89%)	48	24.1
Occasionally (40%–59%)	33	16.6
Rarely (10%–39%)	13	6.5
Almost Never (less than 10%)	6	3.0
Total	199	100.0

**Table 9:** “before recommending altered diet textures, I inform the patient of the possible risks associated with altered diet textures”.

Response	Frequency (*n*)	Percent (%)
Almost Always (90% or more)	79	39.9
Very Frequently (60%–89%)	50	25.3
Occasionally (40%–59%)	44	22.2
Rarely (10%–39%)	16	8.1
Almost Never (less than 10%)	9	4.5
Total	198	100.0

### 3.2 Statistical analysis

Of the 13 total risks associated with MTDs, only three were identified by more than 70% of clinicians: “decreased quality of life” when eating altered texture solids (*n* = 194; 76.7%), “dehydration” when drinking thickened liquids (*n* = 190; 75.1%), and “malnutrition” when eating altered texture solids (*n* = 183; 72.3%). Table 10 details the frequency that each item was selected by study participants.

**Table 10:** Risks associated with altered texture solids and thickened liquids selected by participating SLP*s.*

Altered texture solids	Frequency (*n*)	Percent (%)
Decreased Quality of Life	194	76.7
Malnutrition	183	72.3
Poor recovery from Illness	110	43.5
Dehydration	83	32.8
Thickened Liquids	Frequency (*n*)	Percent (%)
Dehydration	190	75.1
Urinary Tract Infection	151	59.7
Decreased Quality of Life	118	46.6
Constipation	113	44.7
Poor recovery from Illness	99	39.1
Slowed Digestion	2	0.8
Constant Feeling of Thirst	2	0.8
Respiratory Infection	2	0.8
Interfere with Medication Absorption	2	0.8

Two hundred-fifty-three respondents answered questions 11 & 12 for which clinicians were asked to select all known risks associated with MTDs. Ideally, if providing patients with a full accounting of the hazards associated with MTDs, clinicians would correctly identify all 13, but the median score was 8 out of 13. Additionally, more than one in five clinicians (*n* = 55; 21.7%) did not select even a single known risk associated with MTDs, and only 16 respondents (6.3%) were able to correctly identify all 13 risks listed in this survey. Almost one-half of participants (*n* = 126; 49.8%) listed 7 or fewer known complications of MTDs. Table 11 details the frequency of the number of risks selected by SLPs for MTDs. An ANOVA was completed, and none of the demographic categories (e.g., years of experience as an SLP, years of experience treating dysphagia, professional certifications, primary work setting, level of education, formal training, informal training, and number of dysphagia evaluations per week) significantly impacted clinician scores *F*(13, 312) = 4.38, *p* = .234.

**Table 11:** Frequency of risks selected by SLPs for MTD*s.*

Risks Selected	Number of SLPs (*n*=)	Percent (%)
0	55	21.7
1	2	0.8
2	1	0.4
3	6	2.4
4	12	4.7
5	13	5.1
6	20	7.9
7	17	6.7
8	23	9.1
9	18	7.1
10	24	9.5
11	20	7.9
12	26	10.3
13	16	6.3
Total	*n* = 253	100.0

## 4 Discussion

This survey highlights an Achilles’ heel for informed consent that has three key facets. First, the vast majority of participating SLPs, who routinely recommend MTDs when needed, were unable to identify more than half of the known health problems associated with the consumption of MTDs—with a shocking 21.7% unable to select even a single known risk from the list provided. This barrier to gaining informed consent from a patient with OD reveals an even deeper problem for SLPs who work with individuals with dysphagia. Only a very small percentage of SLPs surveyed (6.3%) would be able to fully inform a patient about the known health risks associated with MTDs and compare those risks to the known risks associated with aspiration.

Second, a less-than-optimal number of SLPs reported weighing the known risks of consuming MTDs against the known risks of aspiration. Again, given the requirement to fully inform patients of known risks associated with the use of MTDs, one would hope that all SLPs would select that they “almost always” weigh the known risks associated with MTDs vs. the known risks associated with aspiration. Yet, only 70% (*n* = 140) of participants indicated that they “almost always” weighed the known risks associated with thickened liquids prior to recommending them, and a mere 54% (*n* = 108) of SLPs stated that they “almost always” personally weigh the known risks associated with modified texture solids before recommending their use.

Third, less than half of the SLPs who responded to this survey indicated that they routinely inform their patients of the known risks associated with MTDs. Of the SLPs who took this survey, only 49.7% indicated that they “almost always” inform their patients about the known risks associated with thickened liquids, and only 39.9% of SLPs reported that they “almost always” inform their patients of the known risks associated with altered solid textures. Both numbers are far too low for an intervention that carries serious known health risks. Given these responses, it is likely that a significant portion of SLPs do not realize that full disclosure of these known risks is required for patients to give their informed consent to use of MTDs as an intervention for OD.

Overall, the results from this study appear in line with past surveys of SLP practice patterns. In past surveys regarding management of OD, SLPs consistently demonstrate suboptimal adherence to the use of evidence-based practice related to evaluation and treatment of OD (30, 49–52). However consistent and commonplace these results may be, the SLPs surveyed failed to identify known complications of consuming MTDs—which include malnutrition, dehydration, and dire systemic consequences like poor recovery from illness and respiratory infections. Thus, it appears that SLPs, as a body of clinicians, may not possess the knowledge necessary to adequately inform patients about the health outcomes associated with MTDs.

Diet recommendations made by SLPs require informed consent (27). However, there is ample evidence from multiple well-designed and replicated studies that altering the viscosity of liquids and the texture of solids does not prevent pulmonary complications like pneumonia (7, 15, 16, 20, 29, 32, 34, 53–56). Additionally, as with any medical intervention, there are significant known health risks associated with consuming MTDs (1, 15, 16, 32, 37, 40, 57). Hence, any improvement in airway protection—due to the consumption of MTDs—must be weighed against the known health risks associated with the consumption of MTDs, which include decreased QOL, malnutrition, dehydration, poor recovery from illness, and respiratory infection (20).

Modifying liquid viscosity and modifying solid food textures remains the most common intervention recommended by SLPs for individuals with OD (27). Indeed, the standard practice for treatment of OD appears myopically focused on preventing aspiration at any cost by using MTDs, and this practice has been the primary form of intervention for the last five decades of SLP involvement in OD management (4, 20, 49). Modifying patient diets originated from a desire to have individuals with OD eat and drink with a reduced risk of aspiration. It was hoped that reduced aspiration would decrease rates of pneumonia and lower overall mortality, but MTDs fail to lower rates of pulmonary complications associated with prandial aspiration (20, 58). Moreover, the current survey supports the findings of an already existing body of literature from seminal surveys of clinical practice patterns. Namely, SLPs treating OD, much like other medical professionals (59), exhibit suboptimal levels of evidence-based practice (30, 49–52).

### 4.1 Limitations

This investigation into informed consent for the use of MTDs was not the sole purpose of the original survey from which these data were taken. The research questions for this manuscript were taken from a larger survey of SLP knowledge and training regarding the relationship of malnutrition, dehydration, and pneumonia that occur secondary to OD. Thus, a more specific survey, designed explicitly to answer the current questions at issue, would allow for clearer interpretation and a more nuanced treatment of this very important topic. For example, one of the research questions being examined here is: do SLPs routinely inform their patients with OD of the benefits and known risks associated with MTDs? However, another important question that was not asked, because this study was not designed to specifically answer questions about informed consent, is: do SLPs know that informed consent is required when intervening with MTDs? Understanding whether SLPs, as a whole, know that informed consent is required when recommending the use of MTDs with OD is an important question that was not answered directly by these survey items. Future studies are needed to determine if SLPs know that informed consent is required for the consumption of MTDs.

This study also suffered from some of the classic limitations of electronic surveys. Namely, due to the number of questions needed to answer some larger research questions, our survey had a high number of SLPs who did not finish all of the questions. It is possible that cognitive fatigue impacted the quality of the data received from our participants. Future studies looking into this line of questioning would benefit from a shorter and more focused survey that would limit data errors related to survey fatigue.

### 4.2 Conclusions

These findings have significant implications for practicing SLPs and for medical professionals who treat OD. The literature is clear that MTDs have minimal efficacy when it comes to preventing pulmonary complications like pneumonia. Despite the consensus that MTDs do not prevent pneumonia and other pulmonary complications, SLPs continue to prescribe MTDs as the primary method of preventing or mitigating the risk of chest infections. However, MTDs are associated with serious medical complications like poor recovery from illness, malnutrition, and dehydration—all of which can have dire consequences. The data from this survey indicate that the majority of SLPs are not aware of known complications with MTDs and do not provide a full disclosure of the risks and benefits of MTDs before prescribing them.

## Data Availability

The raw data supporting the conclusions of this article will be made available by the authors, without undue reservation.
