# Supplementary material for: Oropharyngeal dysphagia management and informed consent: a survey of speech-language pathologists’ practice patterns when recommending modified texture diets
Source: Front Rehabil Sci. 2025 May 9;6:1520240. doi: 10.3389/fresc.2025.1520240 (PMC12098351; doi:10.3389/fresc.2025.1520240)
Supplement: Supplementary file 1 [file Datasheet1.docx]

SURVEY QUESTIONS

1. Country

- United States
- Other

2. Age

- 24 or younger
- 25-34
- 35-44
- 45-54
- 55-64
- 65 or older

3. Ethnicity

- Hispanic or Latino
- Not Hispanic or Latino
- Prefer not to answer

4. Race

- American Indian or Alaska Native
- Asian
- Black or African American
- Native Hawaiian or Other Pacific Islander
- White
- Multiracial
- Prefer not to answer

5. What is the highest degree or level of school you have completed? (If currently enrolled, select the highest degree you have received.)

- master's (Master of Arts/Master of Education/Master of Science)
- clinical doctorate (SLPD)
- research doctorate (Doctor of Philosophy/Doctor of Education)

6. How many years have you been an SLP?

- <1
- 1-5
- 6-10
- 11-20
- 21+

7. How many years have you treated individuals with dysphagia?

- <1
- 1-5
- 6-10
- 11-20
- 21+

8. Professional Certifications (Check ALL that apply.)

- Certificate of Clinical Competence (CCC)
- Modified Barium Swallow Impairment Protocol (MBSImP)
- McNeill Dysphagia Therapy Program (MDTP)
- Board Certification Specialist – Swallowing and Swallowing Disorders (BCS-S)
- FEES Competency Training

9. Select your current work setting. (If multiple settings apply, choose the setting you would consider your ***primary*** work environment.)

- Medical hospital
- Rehabilitation hospital
- Pediatric hospital
- Skilled nursing facility
- Home health
- Outpatient
- Private practice
- Research lab
- Long-term care
- Other

10. Including graduate school, continuing education courses, or any other structured training program, have you received formal instruction regarding the relationship between ***dysphagia*** and ***pneumonia***?

- No
- Yes

(For individuals who answered yes, the following text was displayed.) You indicated that you received formal training regarding the relationship between ***dysphagia*** and ***pneumonia***. Please indicate the setting in which you received your training.

- Master's program
- Doctoral program
- Continuing education course
- Other (please specify):

11. Including graduate school, continuing education courses, or any other structured training program, have you received formal instruction regarding the relationship between ***dysphagia*** and ***malnutrition/dehydration***?

- No
- Yes

(For individuals who answered yes, the following text was displayed.) You indicated that you received formal training regarding the relationship between ***dysphagia*** and ***malnutrition/dehydration***. Please indicate the setting in which you received your training.

- Master's program
- Doctoral program
- Continuing education course
- Other (please specify):

12. In a typical week, how many dysphagia evaluations do you perform?

- <1
- 1-2
- 3-5
- 6-10
- 11+

13. Give your best estimate of the percentage of individuals who you evaluated for dysphagia that were referred for flexible endoscopic evaluation of swallowing ***(FEES)*** or modified barium swallow study ***(MBS/VFSS)***?

- 0
- 1-20%
- 21-40%
- 41-60%
- 61-80%
- 81-100%

14. What percentage of your caseload would you estimate involves dysphagia treatment?

- 1-20%
- 21-40%
- 41-60%
- 61-80%
- 81-100%

Survey Questions:

1. From the list provided, select the ***primary goal*** of a ***swallowing evaluation*** completed by a SLP.

- To optimize nutrition and hydration
- To improve quality of life for individuals with dysphagia
- To determine the least restrictive diet
- To develop a treatment plan for rehabilitation or maintenance of swallowing function
- To prevent aspiration
- To prevent pneumonia from aspiration
- To determine the biomechanical deficits associated with dysphagia
- Other:

2. When making recommendations from a swallowing evaluation, the ***primary*** ***goal of dysphagia intervention*** should be:

- To prevent aspiration
- To prevent laryngeal penetration
- To prevent pneumonia
- To optimize nutrition and hydration
- To improve quality of life
- Other:

3. Is the aspiration of food or liquid all that is required for the development of pneumonia?

- No
- Yes

4. What presents a ***greater risk*** to the overall health of an individual with dysphagia?

- Aspiration
- Malnutrition and dehydration
- I don’t know

5. Expressed as a percentage, how often does visualized aspiration result in pneumonia in the patients with ***acute stroke***?

- 1-10%
- 11-25%
- 26-40%
- >40%
- I don’t know

6. Expressed as a percentage, how often does visualized aspiration result in pneumonia in patients who have ***NOT*** ***had an acute stroke***?

- 1-10%
- 11-25%
- 26-40%
- >40%
- I don’t know

7. As part of dysphagia management, I recommend modified diet textures when needed.

- No
- Yes

8. As part of dysphagia management, I recommend thickened liquids when needed.

- No
- Yes

9. Is there a relationship between ***malnutrition*** and the consumption of modified diet textures?

- No
- Yes
- I don’t know

10. Is there a relationship between ***dehydration*** and the consumption of modified diet textures or thickened liquids?

- No
- Yes
- I don’t know

11. From the list below, select ***any risk you know to be associated*** with the consumption of ***modified diet textures***. (Select ALL that apply.)

- Malnutrition
- Dehydration
- Respiratory infection
- Poor recovery from illness
- Constipation
- Urinary tract infection
- Slow digestion
- Interfere with medication absorption
- Decreased quality of life
- Constant feeling of thirst
- None of the items apply
- I don’t know

12. From the list below, select ***any risk you know to be associated*** with the consumption of ***thickened liquids***. (Select ALL that apply.)

- Malnutrition
- Dehydration
- Respiratory infection
- Poor recovery from illness
- Constipation
- Urinary tract infection
- Slow digestion
- Interfere with medication absorption
- Decreased quality of life
- Constant feeling of thirst
- None of the items apply
- I don’t know

13. For a typical individual with dysphagia, I feel it is ***MOST important*** to:

- Eliminate or reduce aspiration
- Improve quality of life
- Improve nutrition and hydration
- Reduce pneumonia risk

14. Before recommending thickened liquids,

I weigh the known risks of aspiration against the known risks of consuming thickened liquids.

- Almost never (less than 10%)
- Rarely (10-39%)
- Occasionally (40-59%)
- Very frequently (60-89%)
- Almost always (90% or more)

I inform the patient of the possible risks associated with thickened liquids.

- Almost never (less than 10%)
- Rarely (10-39%)
- Occasionally (40-59%)
- Very frequently (60-89%)
- Almost always (90% or more)

I inform the medical team (doctors, nurses, dietitians, etc.) of the risks associated with thickened liquids.

- Almost never (less than 10%)
- Rarely (10-39%)
- Occasionally (40-59%)
- Very frequently (60-89%)
- Almost always (90% or more)

15. Before recommending altered diet textures,

I weigh the known risks of aspiration against the known risks of consuming altered diet textures.

- Almost never (less than 10%)
- Rarely (10-39%)
- Occasionally (40-59%)
- Very frequently (60-89%)
- Almost always (90% or more)

I inform the patient of the possible risks associated with altered diet textures.

- Almost never (less than 10%)
- Rarely (10-39%)
- Occasionally (40-59%)
- Very frequently (60-89%)
- Almost always (90% or more)

I inform the medical team (doctors, nurses, dietitians, etc.) of the risks associated with altered diet textures.

- Almost never (less than 10%)
- Rarely (10-39%)
- Occasionally (40-59%)
- Very frequently (60-89%)
- Almost always (90% or more)

16. Indicate the ***primary reason*** that you would recommend an individual consume an altered texture diet.

- Reduced signs/symptoms of aspiration at bedside
- Improvement in overall medical condition
- Improvement in mastication
- Clinical signs of malnutrition or dehydration
- Reduced quality of life
- Improvement in cognition
- Improvement in airway protection on FEES/MBS

17. Indicate the ***primary reason*** that you would recommend upgrading an individual's diet from an altered texture to a regular texture diet.

- Reduced signs/symptoms of aspiration at bedside
- Improvement in overall medical condition
- Improvement in mastication
- Clinical signs of malnutrition or dehydration
- Reduced quality of life
- Improvement in cognition
- Improvement in airway protection on FEES/MBS

18. Indicate the ***primary reason*** that you would recommend an individual consume thickened liquids.

- Signs/symptoms of aspiration at bedside
- Aspiration observed on FEES/MBS
- Patient request
- Reduced oral intake of thin liquids
- Concern for dehydration
- Risk of aspiration
- Risk of pneumonia

19. Indicate the ***primary reason*** that you would recommend upgrading an individual's liquid viscosity from thickened to thin liquids.

- Reduced signs/symptoms of aspiration at bedside
- Improvement in overall medical condition
- Improvement in mastication
- Clinical signs of malnutrition or dehydration
- Reduced quality of life
- Improvement in cognition
- Improvement in airway protection on FEES/MBS
